# Association between Daily-Life Gait Quality Characteristics and Physiological Fall Risk in Older People

**DOI:** 10.3390/s20195580

**Published:** 2020-09-29

**Authors:** Sabine Schootemeijer, Roel H.A. Weijer, Marco J.M. Hoozemans, Kimberley S. van Schooten, Kim Delbaere, Mirjam Pijnappels

**Affiliations:** 1 Department of Human Movement Sciences, Faculty of Behavioural and Movement Sciences, Vrije Universiteit Amsterdam, Amsterdam Movement Sciences, Van der Boechorststraat 7, 1081 BT Amsterdam, The Netherlands; sabine.schootemeijer@radboudumc.nl (S.S.); r.h.a.weijer@LUMC.nl (R.H.A.W.); m.j.m.hoozemans@vu.nl (M.J.M.H.); 2 Department of Neurology, Center of Expertise for Parkinson & Movement Disorders, Donders Institute for Brain, Cognition and Behavior, Radboud University Medical Center, 6500 HB Nijmegen, The Netherlands; 3 Department of Neurology, Leiden University Medical Center, 2300 RC Leiden, The Netherlands; 4 Falls, Balance and Injury Research Centre, Neuroscience Research Australia, University of New South Wales, Sydney, NSW 2031, Australia; k.vanschooten@neura.edu.au (K.S.v.S.); k.delbaere@neura.edu.au (K.D.); 5 School of Public Health and Community Medicine, Faculty of Medicine, University of New South Wales, Sydney, NSW 2033, Australia

**Keywords:** wearable devices, accelerometry, activity monitoring, aged, mobility, locomotion, accidental falls

## Abstract

Gait quality characteristics obtained from accelerometry during daily life are predictive of falls in older people but it is unclear how they relate to fall risk. Our aim was to test whether these gait quality characteristics are associated with the severity of fall risk. We collected one week of trunk accelerometry data from 279 older people (aged 65–95 years; 69.5% female). We used linear regression to investigate the association between six daily-life gait quality characteristics and categorized physiological fall risk (QuickScreen). Logarithmic rate of divergence in the vertical (VT) and anteroposterior (AP) direction were significantly associated with the level of fall risk after correction for walking speed (both *p* < 0.01). Sample entropy in VT and the mediolateral direction and the gait quality composite were not significantly associated with the level of fall risk. We found significant differences between the high fall risk group and the very low- and low-risk groups, the moderate- and very low-risk and the moderate and low-risk groups for logarithmic rate of divergence in VT and AP (all *p* ≤ 0.01). We conclude that logarithmic rate of divergence in VT and AP are associated with fall risk, making them feasible to assess the physiological fall risk in older people.

## 1. Introduction

Falls are a major problem in older people. Every year, approximately one in three people aged 65 year or over falls [1,2]. Previous studies demonstrated that gait quality characteristics obtained from daily life trunk accelerometry discriminate between non-fallers and fallers, both retrospectively and prospectively [3,4,5,6,7,8]. More specifically, sample entropy in vertical and mediolateral direction (gait regularity), the gait quality composite and logarithmic rate of divergence per stride (gait stability, also referred to as the Lyapunov exponent) showed the strongest associations with past and future falls [3,4,5,6,7]. Some but not all of these associations may be a reflection of walking speed, showing better gait quality at higher speeds [9].

Whether gait quality characteristics are associated with fall risk, as opposed to fall incidence or frequency, has not yet been established. The scaling of gait quality measures with measures of fall risk would provide insight into their clinical utility as well as additional evidence on their validity as a fall risk measure. Physiological factors play an important role in determining fall risk and can be assessed by different screening instruments [10]. A brief and validated way to assess physiological fall risk is the QuickScreen score [11], which has been shown to be predictive for multiple falls [12]. This physiological fall risk measure not only takes into account fall history but also includes several measures of someone’s actual physical abilities. Moreover, it is a less noisy outcome measure of fall risk than (accidental) fall incidence [13,14]. For gait quality characteristics to be a valid instrument to identify people who would benefit from fall prevention intervention, these characteristics need to not only be predictive of falls but also associate with physiological fall risk.

Our aim was to examine the association between gait quality characteristics with the severity of physiological fall risk in community-dwelling older people. We expected that, after correcting for walking speed, a better gait quality would be associated with a lower physiological fall risk.

## 2. Materials and Methods

### 2.1. Participants

To investigate whether daily life gait quality characteristics are associated with physiological fall risk, trunk accelerometry data from 279 people aged 65 years or older (69.5% female) who participated in the Veilig in Beweging Blijven (VIBE) study at the Vrije Universiteit Amsterdam, were analyzed. Older people were eligible for participation in the longitudinal VIBE cohort study if they were community-dwelling, able to walk 20 meters with or without walking aids without becoming short of breath, dizzy or perceiving chest pain or pressure, had a Mini Mental Stage Examination (MMSE) score higher than or equal to 19 [15] and were able to understand the Dutch language.

The protocol of the VIBE study was approved by the ethics committee of the faculty of Behavioural and Movement Sciences of the Vrije Universiteit Amsterdam (VCWE-2016-129). All participants provided signed informed consent before participation.

### 2.2. Participants’ Characteristics

Participants’ characteristics were collected during a visit to the laboratory at the Vrije Universiteit, Amsterdam (the Netherlands). Fall history over the 12 months prior to the assessment was obtained with a questionnaire. Participants’ global cognitive function was assessed with the Mini Mental State Examination (MMSE) [15] and the severity of depressive symptoms was assessed with the Geriatric Depression Scale-15 (GDS-15) [16]. Knee extension strength was measured with a unidirectional force transducer (KAP-E 2kN, A.ST. GmbH Dresden, Germany) as validated by Lord and Menz [17]. Handgrip strength was measured with a handgrip dynamometer (TKK 5401, Takei Scientific Instruments, Tokyo, Japan). To determine the maximal knee torque and maximal handgrip strength, participants were instructed to give their maximal effort three times for each leg or hand, respectively. The highest value out of three attempts was considered to be the participant’s maximal effort for that leg or hand. The sum of the maximal knee torque of both legs and the sum of the handgrip strength of both hands was reported.

### 2.3. Assessment of Physiological Fall Risk

Physiological fall risk was estimated with the QuickScreen [11]. This fall risk score has been shown to be predictive for multiple falls (area under the receiver operator curve = 0.72 (95% confidence interval = 0.66–0.79)) [12]. People with two risk factors are at 1.7 times higher risk of future falls compared with people without any risk factors while people exhibiting five or more risk factors are at 8.6 times higher risk of future falls [11]. Participants’ physiological fall risk was determined by the sum of risk factors from eight measures in five different categories (between brackets): history of falls (previous falls), total number of medications and psychoactive medications (medications), visual acuity (vision), tactile sensitivity (peripheral sensation), the sit-to-stand test, near tandem stand test and alternate stepping test (strength, reaction time and balance). Based on the physiological fall risk score, participants were assigned to four subgroups: very low (0 or 1 risk factor), low (2 or 3 risk factors), moderate (4 risk factors) or high (5 or more risk factors) fall risk [11].

### 2.4. Assessment of Daily Life Gait

**Figure 1:**
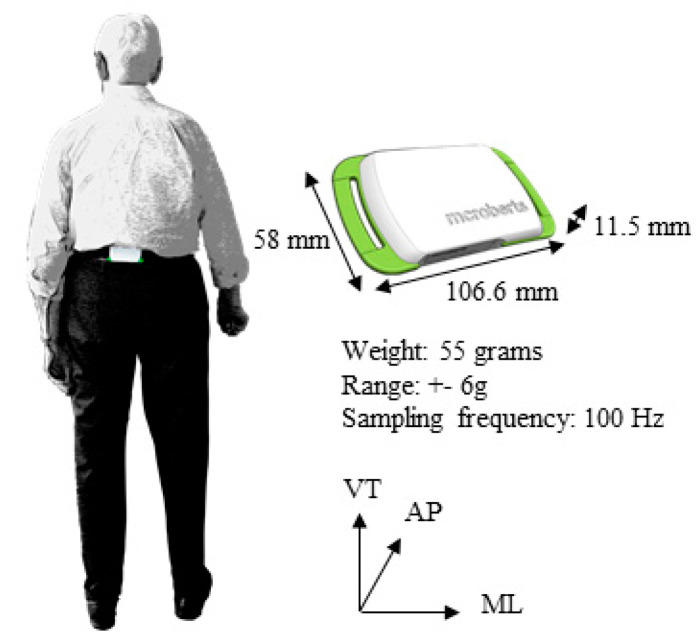
Assessment of daily life gait with the DynaPort MoveMonitor.

Trunk accelerometry data were obtained with a commercially available wearable inertial sensor (DynaPort MoveMonitor, McRoberts, The Hague, The Netherlands). Participants were instructed to wear the inertial sensor on the back of their trunk at belt height with the use of an elastic band (Figure 1). They wore the sensor for one week at all times except during aquatic activities. To exclude data that could have possibly been collected during transportation, the first six hours were excluded from our analysis. The inertial sensor captured accelerations in the vertical (VT), mediolateral (ML) and anteroposterior (AP) direction and had a range of +/− 6 g. The data were set to sample at a frequency of 100 Hz. The sensor’s dimensions were 106.6 × 58 × 11.5 mm and weighed 55 g.

Gait quality characteristics were estimated from trunk accelerometry data using a custom code in MATLAB R2018b (Mathworks, Natrick, MA, USA). For every participant, locomotion episodes that lasted for at least 10 seconds were selected from the acceleration signal using the manufacturer’s algorithm. This classification algorithm was previously validated [18] and showed optimal reliability when measured over at least four consecutive days [19]. The locomotion episodes were divided in epochs of 10 seconds and gait quality characteristics were calculated for each of these epochs. For every gait quality characteristic, we took the median of all epochs over one week as a representation of someone’s daily life gait quality [4,5,7]. In total, we extracted walking speed and six gait characteristics: sample entropy in ML and VT, logarithmic rate of divergence in VT, ML and AP and the gait quality composite [5,14]. Walking speed was determined from the product of stride frequency and stride length, estimated from leg length and vertical trunk displacements assuming compass gait [20]. The gait quality composite is a weighed sum of the root mean square of the acceleration in ML, the index of harmonicity in ML, the magnitude of the acceleration at the dominant period in the frequency domain in the AP direction and the autocorrelation of the acceleration at the dominant period in the frequency domain in VT [14]. The gait quality composite was coded such that a higher gait quality composite score corresponded to a better gait quality. The MATLAB code used to estimate the gait quality composite can be found at github.com/KimvanS/EstimateGaitQualityComposite.

### 2.5. Statistics

Statistical analyses were performed in R (R Core Team (2016), Vienna, Austria). Descriptive characteristics (as dependent variables) were compared between the four fall risk subgroups (as a categorical independent variable) using linear regression, the Kruskal–Wallis test (for age and GDS) and Chi-square (for gender and fall history) analyses. Post hoc pair-wise comparisons were reported with a Bonferroni correction for multiple testing if there was an overall significant effect. Linear regression analyses were performed to investigate whether gait quality characteristics were associated with physiological fall risk. Gait quality characteristics (sample entropy in ML and VT, logarithmic rate of divergence in VT, ML and AP and a gait quality composite score) were treated as dependent variables and physiological fall risk (i.e., the subgroup that people were assigned to with QuickScreen) served as a categorical predictor. Walking speed was included in the linear regression as a covariate.

## 3. Results

### 3.1. Descriptive Characteristics

From the sample of 279 people, 63 older people were classified into the very low-risk group, 165 into the low-risk group, 32 into the moderate-risk group and 19 into the high-risk group. For each subgroup, the mean and standard deviation of the descriptive characteristics are shown in Table 1, unless noted otherwise. People in the higher fall risk groups were older, had a higher body weight, had more often experienced a fall in the previous 12 months, had a lower handgrip strength and knee torque, spent less time walking per day and had a lower walking speed compared with counterparts in lower fall risk groups.

### 3.2. Gait Quality Characteristics after Correction for Speed

There was a main effect of fall risk group on logarithmic rate of divergence in VT (F (3, 273) = 5.50, *p* < 0.01) and logarithmic rate of divergence in AP (F (3, 273) = 6.12, *p* < 0.01). There were no significant main effects of fall risk group on sample entropy VT and AP, gait quality composite score and logarithmic rate of divergence per stride ML (all *p* ≥ 0.05).

The regression coefficients (Table 2) of fall risk severity with the gait quality characteristics revealed significant differences between the high-risk group compared with the very low-risk group (all *p* ≤ 0.01) and the high-risk group compared with the low-risk group (all *p* < 0.05). Logarithmic rate of divergence in VT and AP were significantly different between the moderate-risk group and the very low-risk group (all *p* ≤ 0.01) and the moderate-risk group compared with the low-risk group (all *p* ≤ 0.01). The regression coefficients for the models unadjusted for walking speed are reported in Table A1 (Appendix A).

## 4. Discussion

Our aim was to examine the association between gait quality characteristics with the severity of physiological fall risk in community-dwelling older people. These gait quality characteristics were previously validated on falls but analyses were performed in the same cohort (FARAO cohort, 319 older people aged 65–99 years) [4,5,6,7,14]. In line with the previous validation on falls [5], our results indicated that logarithmic rate of divergence per stride in VT and AP were associated with physiological fall risk while sample entropy and a gait quality composite score were, against our expectations, not associated with physiological fall risk after adjusting for walking speed.

Many gait quality characteristics, including the majority of the selection we evaluated, are dependent on walking speed [5,9]. To correct for walking speed, we included walking speed as a covariate in the linear regression. After the correction for walking speed, logarithmic rate of divergence per stride in VT and AP were, and sample entropy and the gait quality composite were not, significantly associated with physiological fall risk score. The gait quality composite score consists of four characteristics that are dependent on walking speed [5,9] and was just like sample entropy in ML associated with fall risk in the unadjusted analyses (Table A1). It is not surprising that correcting the gait quality composite for walking speed decreased the strength of its association with physiological fall risk, considering the strong negative association between physiological fall risk and walking speed in our cohort (Table 1). As walking speed explains a large part of the associations between daily life gait quality characteristics and falls, it is important to have a reliable estimation of walking speed. We determined walking speed from step frequency and step length, as derived from the vertical trunk displacements [20]. Other methods of estimating walking speed, such as a Global Position System (GPS) [21] or multiple accelerometers may result in even more reliable estimations but may be less easily accepted by users due to privacy concerns or the increased burden of wearing multiple sensors.

We showed that from a comprehensive set of gait quality characteristics, logarithmic rate of divergence in VT and AP were associated with physiological fall risk on a group level. We tested the gait quality characteristics that we considered the best candidates based on previous studies [3,4,5,6,7] and did not aim to test all available gait quality characteristics. The characteristics that we included were validated on falls previously [4,5,6,7,14] but the sensitivity to change to fall prevention interventions remains to be shown.

The novelty of this study is that we investigated the association between gait quality characteristics and physiological fall risk. A high physiological fall risk may promote falls but not if these people are very inactive and do not expose themselves to environmental hazards [4]. On the other hand, falls may also occur accidentally in people with very few physiological fall risk factors but who frequently expose themselves to hazardous situations [22]. Accidental falls are unlikely to be completely predictable by any type of instrument, gait-related or otherwise, hence in the current study we chose physiological fall risk as an outcome measure. As noted, we validated the gait quality characteristics on physiological fall risk (QuickScreen, Tiedemann [11]) rather than falls, which has been done previously [5]. Although the current study provides some evidence for the validity of gait quality characteristics as measures of fall risk, external validation on prospective falls is still needed.

## 5. Conclusions

We conclude that the gait quality characteristics logarithmic rate of divergence in VT and AP are associated with physiological fall risk in older people. These gait quality characteristics appear to be a valid tool to assess fall risk in older people.

## Figures and Tables

**Table 1:** Descriptive characteristics of the total sample and per physiological fall risk group as determined by the QuickScreen test.

Descriptive Characteristics	Total Sample	Very Low-Risk Group	Low-Risk Group	Moderate-Risk Group	High-Risk Group
Number of participants (N (%))	279	63 (22.6%)	165 (59.1%)	32 (11.5%)	19 (6.8%)
Number of females (N (%))	194 (69.5%)	48 (76.2%)	112 (67.9%)	21 (65.6%)	13 (68.4%)
Age (years; Median (IQR))	70.1 (67.6–74.9) *	68.3 (66.9–71.6) ^↓^	69.6 (67.6–73.6) ^↓^	74.5 (69.7–79.0)	78.8 (72.0–82.5)
Body height (cm)	169.0 (8.5)	168.1 (7.6)	169.2 (8.7)	171.7 (8.2)	166.4 (9.3)
Body weight (kg)	74.0 (13.7) *	71.2 (12.6)	73.2 (12.5) ^↔^	79.8 (12.6) ^↑^	80.7 (22.2) ^↑^
At least one fall in the past 12 months (N (%))	136 (48.8%) *	3 (4.8%) ^⇕^	91 (55.2%)	26 (81.3%)	16 (84.2%)
Cognitive function (MMSE score)	31.1 (2.3)	31.5 (1.3)	31.2 (2.6)	30.8 (1.6)	29.9 (2.7)
Depressive symptoms (GDS score; Median (IQR))	5.0 (5.0–6.0)	5.0 (5.0–6.0)	5.0 (5.0–6.0)	5.5 (5.0–6.0)	6.0 (4.5–7.0)
Handgrip strength: females (kg)	50.7 (10.1) *	54.2 (10.2)	50.5 (9.3)	50.1 (8.2)	40.5 (12.6) ^⇕^
Handgrip strength: males (kg)	76.1 (14.1) *	85.3 (12.0) ^↑^	76.9 (13.3) ^↔^	67.2 (14.0)	62.4 (4.0)
Knee torque: females (Nm)	74.8 (25.3) *	84.2 (24.2) ^↑^	75.3 (24.9) ^↔^	60.5 (20.2)	57.8 (24.3)
Knee torque: males (Nm)	98.1 (30.6) *	96.2 (18.0) ^↔^	105.4 (33.6) ^↑^	79.2 (13.4)	69.8 (23.0) ^↔^
Time spent walking (min/day)	83.2 (33.7) *	88.3 (31.3) ^↑^	86.8 (34.6) ^↑^	74.6 (24.6)	49.5 (26.8)
Walking speed (m/s)	0.93 (0.19) *	0.98 (0.21) ^↑^	0.93 (0.18) ^↑^	0.88 (0.21) ^↔^	0.77 (0.13)

Note: Values represent the mean (standard deviations) unless noted otherwise. Handgrip strength and knee torque are reported as left and right combined. * Denotes a significant main effect group (*p*-values below 0.05). Significance level for the pair-wise comparisons after Bonferroni correction was set at *p* = 0.008. ⇕ significantly different from all other groups, ↓ significantly lower than all unmarked, ↑ significantly higher than all unmarked, ↔ not significantly different from other marked group.

**Table 2:** Regression coefficients (β) with standard errors (SE) for the linear regression models adjusted for walking speed.

	Mean Total Sample (SD)	Very Low-Risk Group β (SE), *p*	Low-Risk Group β (SE), *p*	Moderate-Risk Group β (SE), *p*
**Sample entropy VT**	0.24 (0.04)			
Low-risk group		0.00 (0.01), 0.71		
Moderate-risk group		−0.01 (0.01), 0.36	−0.01 (0.01), 0.45	
High-risk group		**−0.03 (0.01), 0.01**	**−0.02 (0.01), 0.01**	−0.02 (0.01), 0.12
**Sample entropy ML**	0.34 (0.05)			
Low-risk group		−0.01 (0.01), 0.35		
Moderate-risk group		−0.02 (0.01), 0.08	−0.01 (0.01), 0.20	
High-risk group		**−0.03 (0.01), 0.01**	**−0.03 (0.01), 0.03**	−0.01 (0.01), 0.32
**Gait quality Composite Score (Z)**	0.71 (0.88)			
Low-risk group		0.03 (0.08), 0.71		
Moderate-risk group		−0.11 (0.12), 0.37	−0.14 (0.11), 0.20	
High-risk group		0.17 (0.15), 0.26	0.14 (0.14), 0.31	0.28 (0.16), 0.09
**Log rate of divergence per stride VT**	1.63 (0.39)			
Low-risk group		0.03 (0.03), 0.38		
Moderate-risk group		**0.14 (0.05), <0.01**	**0.11 (0.04), 0.01**	
High-risk group		**0.19 (0.06), <0.01**	**0.17 (0.06), <0.01**	0.06 (0.07), 0.39
**Log rate of divergence per stride ML**	1.96 (0.31)			
Low-risk group		0.03 (0.04), 0.44		
Moderate-risk group		0.09 (0.06), 0.14	0.06 (0.05), 0.27	
High-risk group		**0.19 (0.07), 0.01**	**0.16 (0.07), 0.02**	0.10 (0.08), 0.21
**Log rate of divergence per stride AP**	1.79 (0.31)			
Low-risk group		0.04 (0.03), 0.25		
Moderate-risk group		**0.15 (0.05), <0.01**	**0.11 (0.04), 0.01**	
High-risk group		**0.22 (0.06), <0.01**	**0.18 (0.06), <0.01**	0.07 (0.07), 0.32

Note: Bold values are significant coefficients. Mean (SD) of the total sample reported.

## Appendix A

**Table A1:** Regression coefficients (β) with standard errors (SE) for the linear regression models adjusted for walking speed.

	Very Low-Risk Group	Low-Risk Group	Moderate-Risk Group
	Unadjusted β (SE), *p*	Unadjusted β (SE), *p*	Unadjusted β (SE), *p*
**Sample entropy VT**			
Low-risk group	0.00 (0.01), 0.73		
Moderate-risk group	0.00 (0.01), 0.91	0.00 (0.01), 0.89	
High-risk group	−0.01 (0.01), 0.47	−0.01 (0.01), 0.32	−0.01 (0.01), 0.46
**Sample entropy ML**			
Low-risk group	−0.01 (0.01), 0.32		
Moderate-risk group	−0.02 (0.01), 0.06	−0.01 (0.01), 0.18	
High-risk group	**−0.03 (0.01), 0.01**	**−0.03 (0.01), 0.02**	−0.01 (0.01), 0.29
**Gait Quality Composite Score (Z)**			
Low-risk group	−0.15 (0.13), 0.26		
Moderate-risk group	**−0.48 (0.19), 0.01**	**−0.33 (0.17), 0.05**	
High-risk group	**−0.57 (0.23), 0.01**	**−0.42 (0.21), 0.05**	−0.09 (0.25), 0.73
**Log rate of divergence per stride VT**			
Low-risk group	0.11 (0.05), 0.05		
Moderate-risk group	**0.30 (0.08), <0.01**	**0.19 (0.07), 0.01**	
High-risk group	**0.51 (0.1), <0.01**	**0.41 (0.09), <0.01**	**0.21 (0.11), 0.04**
**Log rate of divergence per stride ML**			
Low-risk group	0.06 (0.04), 0.15		
Moderate-risk group	**0.16 (0.06), 0.02**	0.09 (0.06), 0.11	
High-risk group	**0.32 (0.08), <0.01**	**0.26 (0.07), <0.01**	**0.17 (0.09), 0.05**
**Log rate of divergence per stride AP**			
Low-risk group	**0.09 (0.04), 0.04**		
Moderate-risk group	**0.25 (0.06), <0.01**	**0.16 (0.06), <0.01**	
High-risk group	**0.41 (0.08), <0.01**	**0.33 (0.07), <0.01**	**0.16 (0.08), 0.05**

Note: Bold values are significant coefficients.
